# Risk of metastatic disease using [^18^F]PSMA-1007 PET/CT for primary prostate cancer staging

**DOI:** 10.1186/s13550-021-00869-5

**Published:** 2021-12-20

**Authors:** Venkata Avinash Chikatamarla, Satomi Okano, Peter Jenvey, Alexander Ansaldo, Matthew J. Roberts, Stuart C. Ramsay, Paul A. Thomas, David A. Pattison

**Affiliations:** 1 Department of Nuclear Medicine and Specialised PET Services, Royal Brisbane and Women’s Hospital, Brisbane, QLD 4006 Australia; 2 Faculty of Medicine, University of Queensland, Brisbane, Australia; 3 Statistics Unit, QIMR Berghofer Medical Research Institute, Brisbane, Australia; 4 Department of Medical Imaging, Royal Brisbane and Women’s Hospital, Brisbane, Australia; 5 Department of Urology, Royal Brisbane and Women’s Hospital, Brisbane, Australia; 6 Department of Urology, Redcliffe Hospital, Redcliffe, Australia; 7 Centre for Clinical Research, The University of Queensland, Brisbane, Australia; 8 School of Medicine, James Cook University, Townsville, Australia

**Keywords:** [^18^F]PSMA-1007 PET/CT, Staging, Prostate cancer, Metastases, SUVmax, PSA, ISUP grade

## Abstract

**Background:**

Accurate prostate cancer imaging is critical for patient management. Multiple studies have demonstrated superior diagnostic accuracy of [^68^Ga]-PSMA-11 PET/CT over conventional imaging for disease detection, with validated clinical and biochemical predictors of disease detection. More recently [^18^F]PSMA-1007 offers theoretical imaging advantages, but there is limited evidence of clinical and biochemical predictors of scan findings in the staging population. This study investigates the association of clinical variables with imaging characteristics among patients who underwent [^18^F]PSMA-1007 PET/CT for primary staging of men with histopathologically confirmed prostate carcinoma. A retrospective review of 194 consecutive patients imaged between May 2019 to May 2020 was performed. Association between imaging variables (presence and distribution of metastatic disease, primary tumour SUVmax) and clinical variables (EAU risk criteria) were assessed using descriptive statistics, logistic regression model and ROC analysis.

**Results:**

The median age, PSA level and ISUP grade were 70 years, 10 ng/mL and ISUP grade 3, respectively. There were 36.6% of patients with intermediate-risk and 60.8% of patients with high-risk disease. ISUP grade was associated with the presence of metastasis overall (*p* = 0.008) as well as regional nodal (*p* = 0.003), non-regional nodal (*p* = 0.041) and bone (*p* = 0.006) metastases. PSA level was associated with metastatic disease overall (*p* = 0.001), regional (*p* = 0.001) and non-regional nodal metastases (*p* = 0.004), but not with bone metastases (*p* = 0.087). There were too few visceral metastases for meaningful analysis. SUVmax of the primary prostatic tumour was associated with ISUP grade (*p* = 0.004), PSA level (*p* < 0.001) and AJCC stage (*p* = 0.034). PSA > 20 ng/mL and ISUP grade > 3 had a specificity of 85% (95% CI 78–91%) and 60% (95% CI 50–68%) and a sensitivity of 36% (95% CI 25–49%) and 62% (95% CI 49–74%), respectively, for detection of metastatic disease.

**Conclusion:**

Metastatic disease according to [^18^F]PSMA-1007 PET/CT was associated with ISUP grade and PSA level. This is the largest study using [^18^F]PSMA-1007 PET/CT to confirm a positive correlation of PSA level, ISUP grade and stage with primary prostate tumour SUVmax.

**Supplementary Information:**

The online version contains supplementary material available at 10.1186/s13550-021-00869-5.

## Background

Prostate cancer is the second most commonly diagnosed malignancy worldwide among males after lung cancer, leading to a significant burden on health systems globally [1, 2]. Hence, accurate staging of newly diagnosed cases is crucial for patient management. However, traditional staging techniques such as computed tomography (CT) and bone scintigraphy imaging have relatively low sensitivity for early and small volume disease detection [3].

Prostate specific radiotracers that bind to prostate-specific membrane antigen (PSMA), a type II transmembrane glycoprotein strongly overexpressed in prostate cancer cells, have revolutionised prostate cancer imaging and diagnosis. PSMA PET/CT is a sensitive imaging technique with superior diagnostic accuracy over conventional imaging (CT and bone scan) for staging prostate cancer [4, 5]. PSMA PET/CT has a higher sensitivity (97%) and lower specificity (66%) compared to multiparametric MRI (mpMRI) (sensitivity 87% and specificity 68%) and choline PET/CT (sensitivity 73% and specificity 88%) for initial staging of prostate cancer [6, 7]. With biochemical recurrence, PSMA PET/CT has a higher disease detection rate than choline PET/CT and mpMRI. The detection rates for PSMA PET/CT and choline PET/CT stratified by PSA level are 50.0% and 35.0% for PSA level < 0.5 ng/mL; 62.8% and 41.0% for PSA level 0.5–0.99 ng/mL; 73.1% and 62.0% for PSA level 1.0–1.99 ng/mL; 91.7% and 80.0% for PSA level ≥ 2 ng/mL, respectively [8, 9]. Detection rates for mpMRI are 31.3% for PSA < 1 ng/mL and 55.6% for PSA > 1 ng/mL [10].

PSMA can be coupled with either Gallium-68 (^68^Ga) and Fluorine-18 (^18^F) for diagnostic imaging. [^68^Ga]Ga-PSMA-11 is currently the most widely used radiotracer for prostate cancer imaging [11]. However, ^68^Ga-labelled compounds have both cost and logistical limitations for scanning a large volume of patients, leading to the development of ^18^F-labelled PSMA radiotracers [12]. Advantages of ^18^F-labelled radiotracers include: (1) longer half-life (110 min vs 68 min for ^68^Ga) and cyclotron production, facilitating centralised production and distribution leading to reduced costs, and (2) lower positron energy of ^18^F compared to ^68^Ga resulting in better intrinsic spatial resolution. Minimal urinary radiotracer excretion is an additional advantage specific to [^18^F]PSMA-1007 over [^68^Ga]Ga-PSMA-11. Early studies have shown [^18^F]PSMA-1007 PET/CT to have excellent disease detection efficiency comparable and potentially superior to [^68^Ga]Ga-PSMA-11 PET/CT [13, 14].

In clinical practice, risk stratification tools such as D'Amico Risk Classification for Prostate Cancer and Memorial Sloan Kettering Cancer Center (MSKCC) Pre-Radical Prostatectomy nomogram use Prostate-specific antigen (PSA) level, Gleason score and clinical stage to stratify patients as low, intermediate or high-risk for prognostication and management [15]. European Association of Urology (EAU) has adopted the risk stratification used by D'Amico [16, 17]. Several studies have demonstrated the correlation between prognostic risk factors such as PSA level and Gleason score with metastatic disease extent [18–23] and SUVmax [24, 25] of the primary tumour using Ga68-PSMA PET/CT. However, this correlation has not been confirmed with findings on [^18^F]PSMA-1007 PET/CT.

This study aims to investigate the correlation of PSA level and International Society of Urological Pathology (ISUP) grade with the presence and distribution of metastatic disease and SUVmax of the primary prostate tumour using [^18^F]PSMA-1007 PET/CT in staging of patients with histopathologically confirmed prostate carcinoma. Our study also aims to evaluate the EAU high-risk criteria (PSA level > 20 ng/mL or ISUP grade > 3) in the detection of metastatic disease using [^18^F]PSMA-1007 tracer in our population.

## Methods

### Study design and technique

**Fig. 1:**
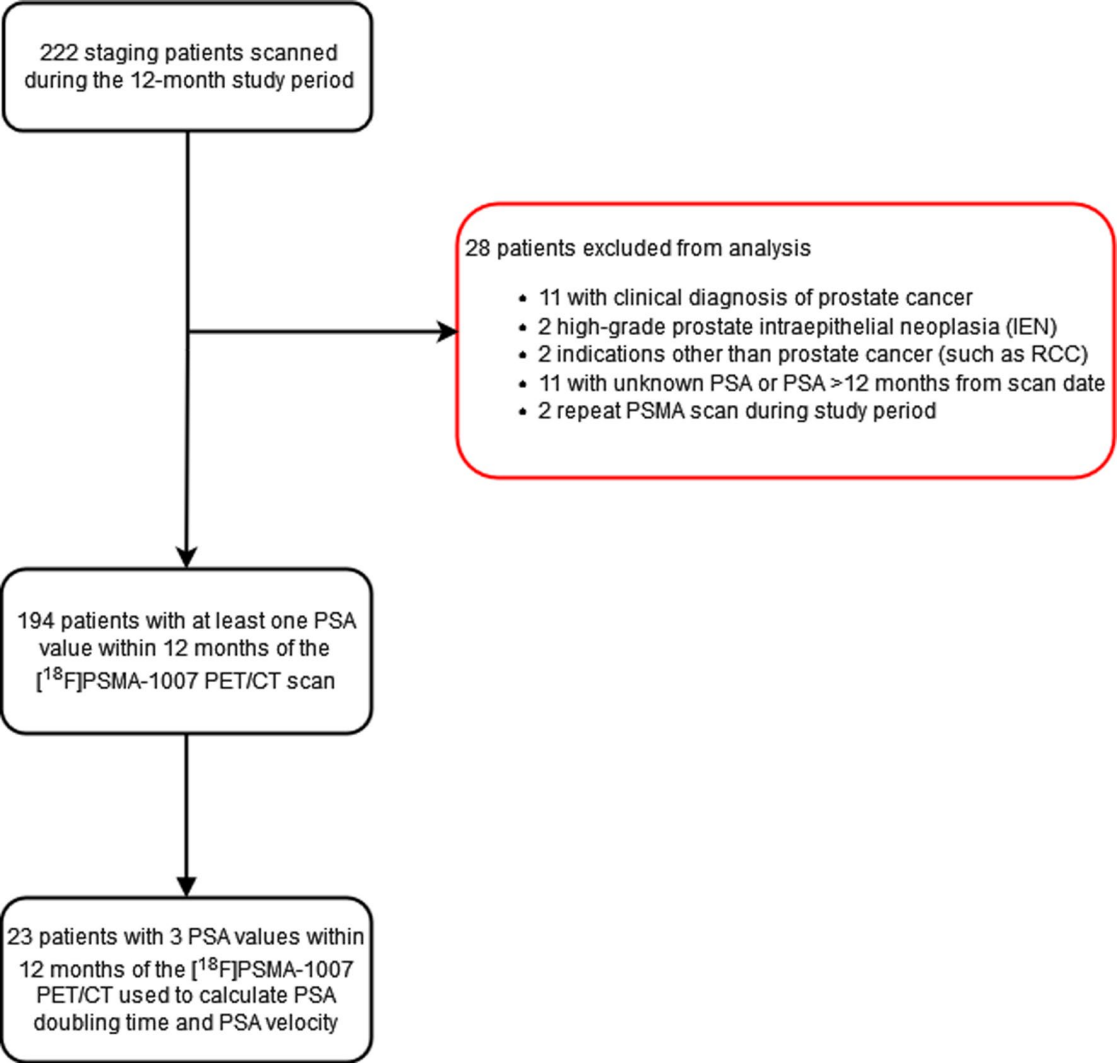
Patient selection flowchart. PSA = Prostate specific antigen; PET/CT = Positron Emission Tomography/Computed Tomography

A retrospective audit of 194 consecutive patients who underwent [^18^F]PSMA-1007 PET/CT scan for initial staging of biopsy proven prostate carcinoma at Royal Brisbane and Women's Hospital (RBWH), a tertiary referral centre, over 12 months between May 2019 to May 2020 was performed. Analysis of the first [^18^F]PSMA-1007 PET/CT was performed if multiple scans were undertaken during the study period. Referral criteria for scanning included PSA > 20 ng/mL or ISUP grade ≥ 3 or clinical stage ≥ T2c. Additionally, at least one PSA value within 12 months preceding the [^18^F]PSMA-1007 PET/CT scan was required for inclusion in the study (Fig. 1). However, 31 patients did not meet the referral criteria (i.e. ISUP ≤ 2 or PSA < 20 or clinical stage < T2c) but were scanned for other reasons (e.g. high-risk radiological features, perineural invasion on biopsy etc.).

Demographic, clinical, biochemical and radiological variables were collected using the electronic medical record systems.

A low-risk ethics exemption was obtained for this study from the Royal Brisbane and Women's Hospital Human Research Ethics Committee (HREC) with a waiver of written consent for this retrospective study.

### [^18^F]PSMA-1007 Radiosynthesis and Quality Control

On-site synthesis of [^18^F]PSMA-1007 was performed using a kit-based approach on GE FASTlab or MX Tracerlab platforms, followed by comprehensive quality control testing to pharmacopoeia standards for other F-18 radiopharmaceuticals (i.e. radionuclide, radiochemical and chemical purity, endotoxin and sterility, pH). The [^18^F]PSMA-1007 radiochemical purity was > 95% as measured using high-performance liquid chromatography (HPLC).

### Imaging protocol

All [^18^F]PSMA-1007 PET/CT scans were performed at the RBWH Nuclear Medicine and Specialised PET Service department on one of three Siemens Healthineers PET/CT (Biograph Vision, Biograph mCT or Biograph mCT Flow) scanners. [^18^F]PSMA-1007 was administered intravenously (mean activity 250 MBq, range 133–284 MBq), with patients being scanned after a median imaging delay of 127 min (IQR 120–140 min). A low-dose non-contrast CT was also performed from the vertex to mid-thighs for attenuation correction and anatomical localisation. PET acquisition was performed in three-dimensional mode (acquisition time of 4-min per bed position over the pelvis and 2.5 min bed position over rest of the imaged body) with emission data corrected for attenuation, scatter, randoms and dead time. The corrected emission data were iteratively reconstructed using ordered-subsets expectation maximisation (3 iterations, 21 subsets) with time of flight and point-spread function resolution recovery, followed by a post-reconstruction Gaussian filter.

### Imaging analysis

All the [^18^F]PSMA-1007 PET/CT scans were dual-reported by two experienced nuclear medicine specialists. The images were reviewed for reporting in Syngio.via (VB50, Siemens Software) which allows CT, PET and fused images to be viewed in multiple planes. Focal [^18^F]PSMA-1007 avidity above background with typical appearance of prostate cancer (with or without definitive anatomic correlate on CT) and distribution not correlating to physiologic uptake (such as cervical, celiac or sacral ganglia) or non-specific bone uptake (obvious indolent bone lesions, degenerative change or fractures) were defined as metastatic disease. Any disagreement was resolved by consensus with a third nuclear medicine specialist. Equivocal lesions (predominantly bone uptake) were not considered malignant.

### Standard of reference

We use [^18^F]PSMA-1007 PET/CT as a standard of reference instead of histopathological validation. This is based on strong evidence with multiple studies using both [^18^F]PSMA-1007 PET/CT and [^68^Ga]Ga-PSMA-11 PET/CT confirming an excellent diagnostic accuracy and high positive predictive value for the detection of metastatic disease with histopathological validation [4, 13, 14, 26–29]. Additionally, a recent prospective intra-individual comparative study has demonstrated excellent concordance between [^18^F]PSMA-1007 and [^68^Ga]Ga-PSMA-11 PET/CT [30, 31]. Therefore, we consider [^18^F]PSMA-1007 PET/CT as an acceptable substitute for histopathological validation based on this extensive literature.

### Statistical analysis

Patient characteristics were summarised using count and percentage for categorical measures and mean and standard deviation (SD) or median and interquartile range (IQR) for continuous measurements. Logistic regression was performed to examine the association between pre-selected patient clinical characteristics including patient age, PSA level, ISUP grade and ADT exposure and scan outcomes including presence or absence of metastatic disease. For multivariable modelling, the variables with *p* < 0.20 in univariable analyses were included. The ISUP grades were grouped during analysis according to the EAU risk groups that use PSA level, ISUP grade/Gleason score (GS) and clinical stage to stratify patients. Low-risk is defined as PSA < 10 ng/mL and ISUP grade 1/GS < 7 and cT1-2a, intermediate-risk as PSA 10–20 ng/mL or ISUP grades 2 and 3/GS 7 or cT2b and high-risk as PSA > 20 ng/mL or ISUP grades 4 and 5/GS > 7 or cT2c or higher. As the clinical stage data were unavailable, patients were grouped into stage IIIb or below, IIIc, IVa and IVb according to American Joint Committee on Cancer **(**AJCC) stage (8th edition, 2017).

SUVmax of the primary prostatic tumour was compared among groups based on PSA level, ISUP grade or AJCC stage using Kruskal–Wallis or Mann–Whitney U test, and the proportion of metastases were compared using Chi-squared test or Fisher’s exact test when more than 20% of the expected counts were less than 5.

The sensitivity and specificity of EAU high-risk criteria was examined using [^18^F]PSMA-1007 PET/CT scan outcome to detect metastatic disease. Median PSA doubling time (Dt) was obtained, including patients who had decreasing PSA (negative PSA doubling time) by using the slope on logged PSA (i.e. log(2)/Dt) [32]. The level of statistical significance was set at 0.05. STATA 15 (StataCorp. 2017. Stata Statistical Software: Release 15. College Station, TX: StataCorp LLC) and R (R version 4.1.0) were used for statistical analysis and graphs.

## Results

### Patient characteristics

A total of 194 patients were included in the final analysis (Table 1). The median age at the time of PET/CT was 70 years (IQR 63–75). The median PSA level prior to the PET/CT was 10 ng/mL (IQR 6.5–18). Using the EAU risk stratification system, 71 (36.6%) patients were classified as intermediate-risk (25.8% of patients with PSA 10–20 ng/mL or 48.2% of patients with ISUP grades 2 and 3), and 118 (60.8%) patients were classified as high-risk (22.2% of patients with PSA > 20 ng/mL or 47.6% of patients with ISUP grades 4 and 5). Further, 7.2% of patients had exposure to ADT prior to the PET/CT with a median of 2 months (IQR 2–5) duration of ADT exposure. Table 2 demonstrates the distribution of PSA levels within each ISUP grade in our imaged population.

**Table 1:** Patient characteristics (*n* = 194)

	Total
	*n* = 194
Age at time of PET/CT (years), median (IQR)	70.0 (63.0–75.0)
Most recent PSA prior to PET/CT (ng/mL), median (IQR)	10.0 (6.5–18.0)
PSA groups (ng/mL) (n = 194)	
≤10	101 (52.1%)
10 –20	50 (25.8%)
> 20	43 (22.2%)
ISUP grade (n = 189)	
1	8 (4.2%)
2	41 (21.7%)
3	50 (26.5%)
4	34 (18.0%)
5	56 (29.6%)
Previous/current ADT prior to the PET/CT scan	14 (7.2%)
Duration of ADT prior to PET/CT scan (months), median (IQR) (n = 14)	2.0 (2.0–5.0)

Data presented as median (IQR) for continuous variables and *n* (%) for categorical variables; n = number; ADT = Androgen Deprivation Therapy; IQR = interquartile range

**Table 2:** Distribution of PSA level within each ISUP grade group (n = 189)

	PSA (ng/mL)		
	≤ 10	10–20	> 20
ISUP grade (n *=* 189)			
1	3 (38%)	2 (25%)	3 (38%)
2	23 (56%)	12 (29%)	6 (15%)
3	26 (52%)	8 (16%)	16 (32%)
4	19 (56%)	9 (26%)	6 (18%)
5	29 (52%)	18 (32%)	9 (16%)
Unknown^a^	1 (20%)	1 (20%)	3 (60%)

*n*(%) = number of patients (% of patients in ISUP grade group)

^a^Five patients had missing ISUP grade information

### ISUP grade & PSA level and metastasis risk

Overall, metastatic disease (regional and distant) was detected in 34% (66/194) of the population. ISUP grade data were unavailable for five patients resulting in 189 patients used for ISUP grade analysis. Metastases were detected in 13% (1/8) with ISUP grade 1 (low-risk), 25% (23/91) with ISUP grades 2 and 3 (intermediate-risk) and 43% (39/90) with ISUP grades 4 and 5 (high-risk). The ISUP grades 4 and 5 have an increased likelihood of metastasis compared to ISUP grades 2 and 3 (OR 2.26; 95% CI 1.20–4.25) and ISUP grade 1 (OR 5.35; 95% CI 0.63–45.3) (Table 3).

**Table 3:** Association of ISUP grade (n = 189), PSA level (n = 194) and ADT (n = 14) with the presence/absence of metastatic disease

	No metastasis	Metastasis present	Univariable model^a^		Multivariable model^a^ (n = 189)	
			Unadjusted OR (95%CI)	*p*-value	Adjusted OR (95%CI)	*p*-value
	*n* = 128	*n* = 66				
Age (years), mean (SD)	68.9 (7.5)	69.9 (8.6)	1.02 (0.98,1.06)	0.39		
ISUP Grade				0.014		0.008
ISUP 1	88% (7/8)	13% (1/8)	0.42 (0.05,3.62)		0.32 (0.03,3.00)	
ISUP 2 and 3	75% (68/91)	25% (23/91)	(reference)		(reference)	
ISUP 4 and 5	57% (51/90)	43% (39/90)	2.26 (1.20,4.25)*		2.64 (1.34,5.23)**	
PSA levels (ng/mL)				0.004		0.001
$\le$ 10	71.3% (72/101)	28.7% (29/101)	(reference)		(reference)	
10–20	74.0% (37/50)	26.0% (13/50)	0.87 (0.41,1.87)		0.93 (0.42,2.07)	
> 20	44.2% (19/43)	55.8% (24/43)	3.14 (1.50,6.58)**		4.33 (1.89,9.91)***	
Previous or current ADT use				0.198		0.198
No	67.2% (121/180)	32.8% (59/180)	(reference)		(reference)	
Yes	50.0% (7/14)	50.0% (7/14)	2.05 (0.69,6.12)		2.16 (0.67,6.96)	

^*^p < 0.05; **p < 0.01; ***p < 0.001

^a^binary logistic regression

**Fig. 2:**
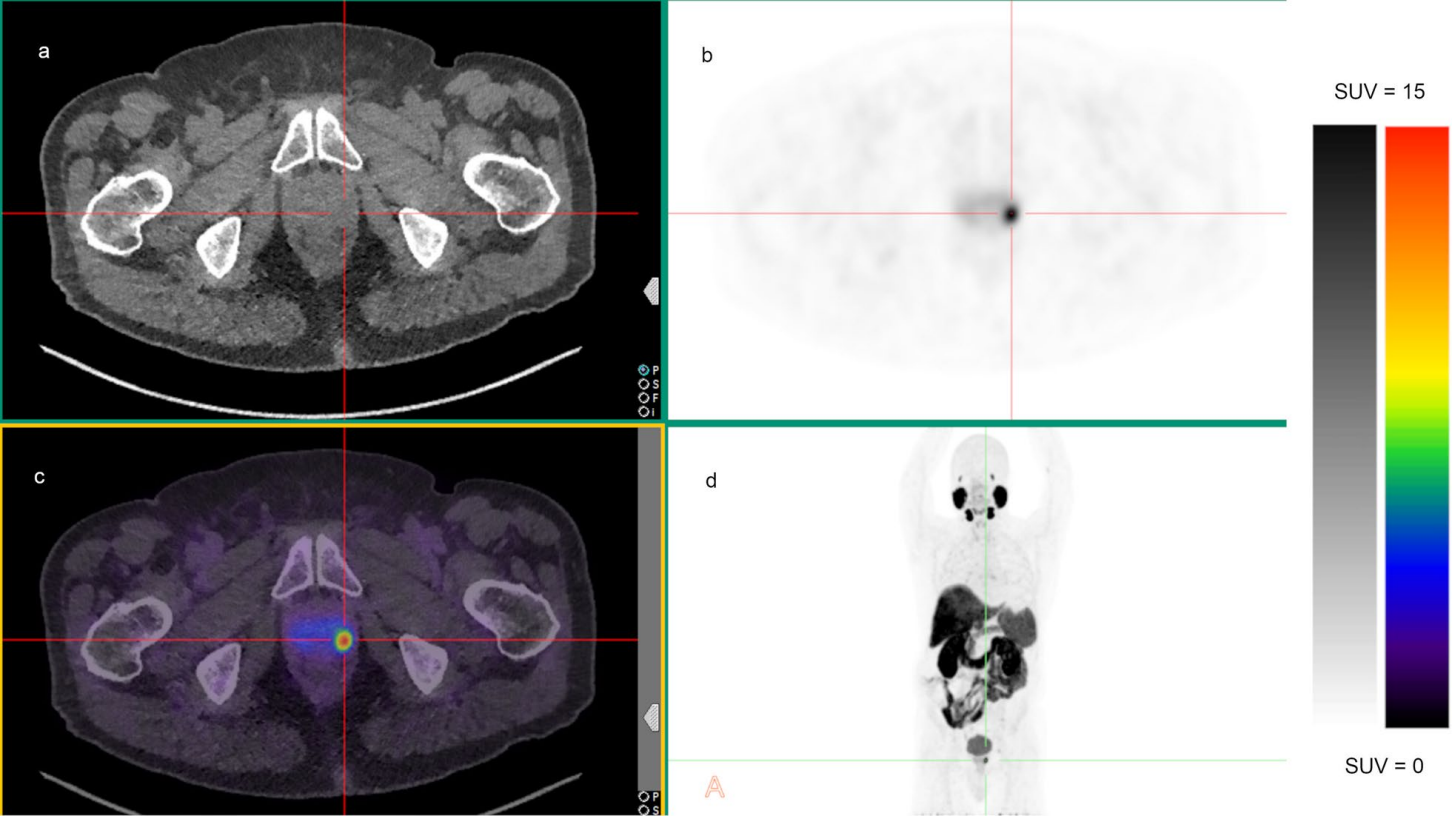
A 70-year-old male with unfavourable intermediate-risk prostate cancer (ISUP grade 3; PSA 5.2 ng/mL) who underwent [^18^F]PSMA-1007 PET/CT that showed a solitary F-PSMA avid focus within the left mid posterolateral prostate gland (SUVmax 14.9). **a** Axial non-contrast CT, **b** axial PET attenuation corrected image and **c** axial fused PET/CT image at the level of the prostate gland. **d** Maximum intensity projection (MIP) image

**Fig. 3:**
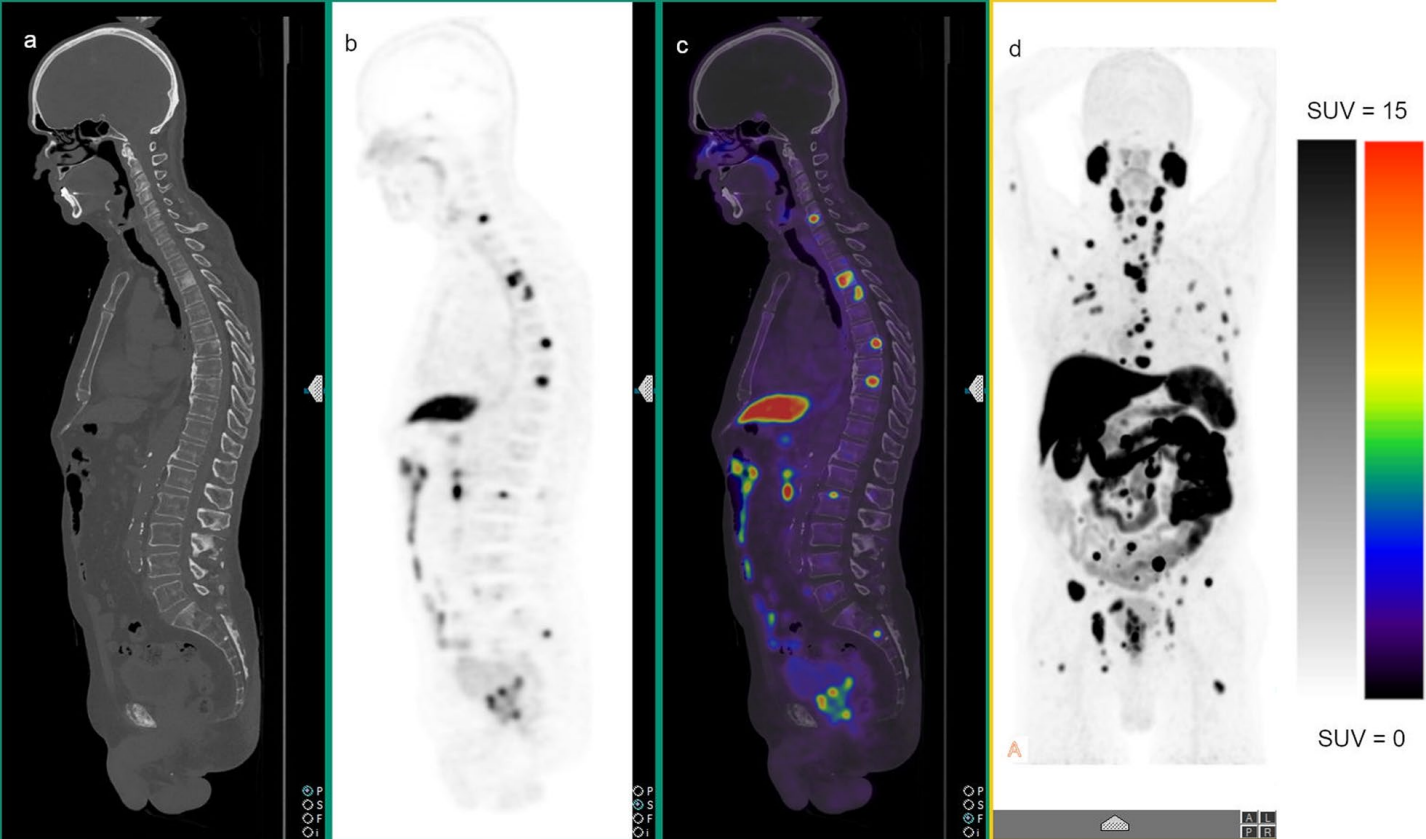
An 88-year-old male with high-risk prostate cancer (ISUP grade 4; PSA 142 ng/mL) underwent [^18^F]PSMA-1007 PET/CT demonstrating intensely F-PSMA avid primary tumour (SUVmax 17.8) and multifocal F-PSMA avid distant metastatic disease. **a** Sagittal non-contrast CT image on bone window, **b** sagittal PET attenuation correction image and **c** sagittal fused PET/CT image from the vertex to pelvis. **d** Maximum intensity projection (MIP) image

Metastases were detected in 28.7% (29/101) with PSA $\le$ 10 ng/mL, 26% (13/50) with PSA 10–20 ng/mL and 55.8% (24/43) with PSA > 20 ng/mL. Patients with PSA levels > 20 ng/mL were more likely to have metastasis, compared to those with PSA levels 10–20 ng/mL (OR 3.60; 95%CI 1.50–8.60) and PSA levels ≤ 10 ng/mL (OR 3.14; 95% CI 1.50–6.58; Table 3). Multivariable modelling including ISUP grade, PSA level and ADT exposure demonstrated association of ISUP grade and PSA level with the presence of metastatic disease (*p* = 0.008 and *p* = 0.001, respectively) such that patients with higher ISUP grade and PSA level were more likely to have metastatic disease (Table 3). Although a higher proportion of metastatic disease was detected in patients with previous or current use of ADT prior to the PET/CT scan (50% vs. 33%; aOR 2.16, 95%CI 0.67–6.96), this was not statistically significant. Figures 2 and 3 show examples of [^18^F]PSMA-1007 PET/CT with localised prostate disease and extensive metastases, respectively.

### ISUP grade & PSA level and distribution of metastasis

Overall, lymph node/nodal (regional and non-regional) metastases were detected in 22.2% (43/194), bone metastases were detected in 20.6% (40/194), and visceral metastases were detected in 3.6% (7/194) of patients. There were 12.5% (1/8) regional nodal metastases in ISUP grade 1, 11% (10/91) in ISUP grades 2 and 3 and 31% (28/90) in ISUP grades 4 and 5. ISUP grade 1 had no non-regional nodal, bone or visceral metastasis. ISUP grades 2 and 3 had 4.4% (4/91) non-regional nodal, 12.1% (11/91) bone and 3.3% (3/91) visceral metastases whereas ISUP grades 4 and 5 had 14.4% (13/90) non-regional nodal, 28.9% (26/90) bone and 4.4% (4/90) visceral metastases. There was significant positive association between ISUP grade and the presence of regional nodal (*p* = 0.003), non-regional nodal (*p* = 0.041) and bone (*p* = 0.006) metastases. There were too few visceral metastases for meaningful analysis (*p* = 0.780) (Table 4).

**Table 4:** Association of ISUP grade with SUVmax of the primary prostatic tumour and distribution of metastasis on [^18^F]PSMA-1007 PET/CT

	ISUP grade groups			*p*-value
	1	2 and 3	4 and 5	
	*n* = 8	*n* = 91	*n* = 90	
SUVmax of primary tumour (*n* = 183), median (IQR)^a^	8.4 (5.2–15.2)	14.9 (8.8–24.3)	19.6 (11.9–32.3)	0.004
Regional nodes (*n* = 189), %(n/Total)^b^	12.5% (1/8)	11.0% (10/91)	31.1% (28/90)	0.003
Non-regional nodes (*n* = 189), %(*n*/Total)^b^	0% (0/8)	4.4% (4/91)	14.4% (13/90)	0.041
Bone metastasis (*n* = 189), %(*n*/Total) ^b^	0% (0/8)	12.1% (11/91)	28.9% (26/90)	0.006
Visceral metastasis (*n* = 189), %(*n*/Total) ^b^	0% (0/8)	3.3% (3/91)	4.4% (4/90)	0.78

^a^Kruskal-Wallis-test

^b^Chi-squared test

**Fig. 4:**
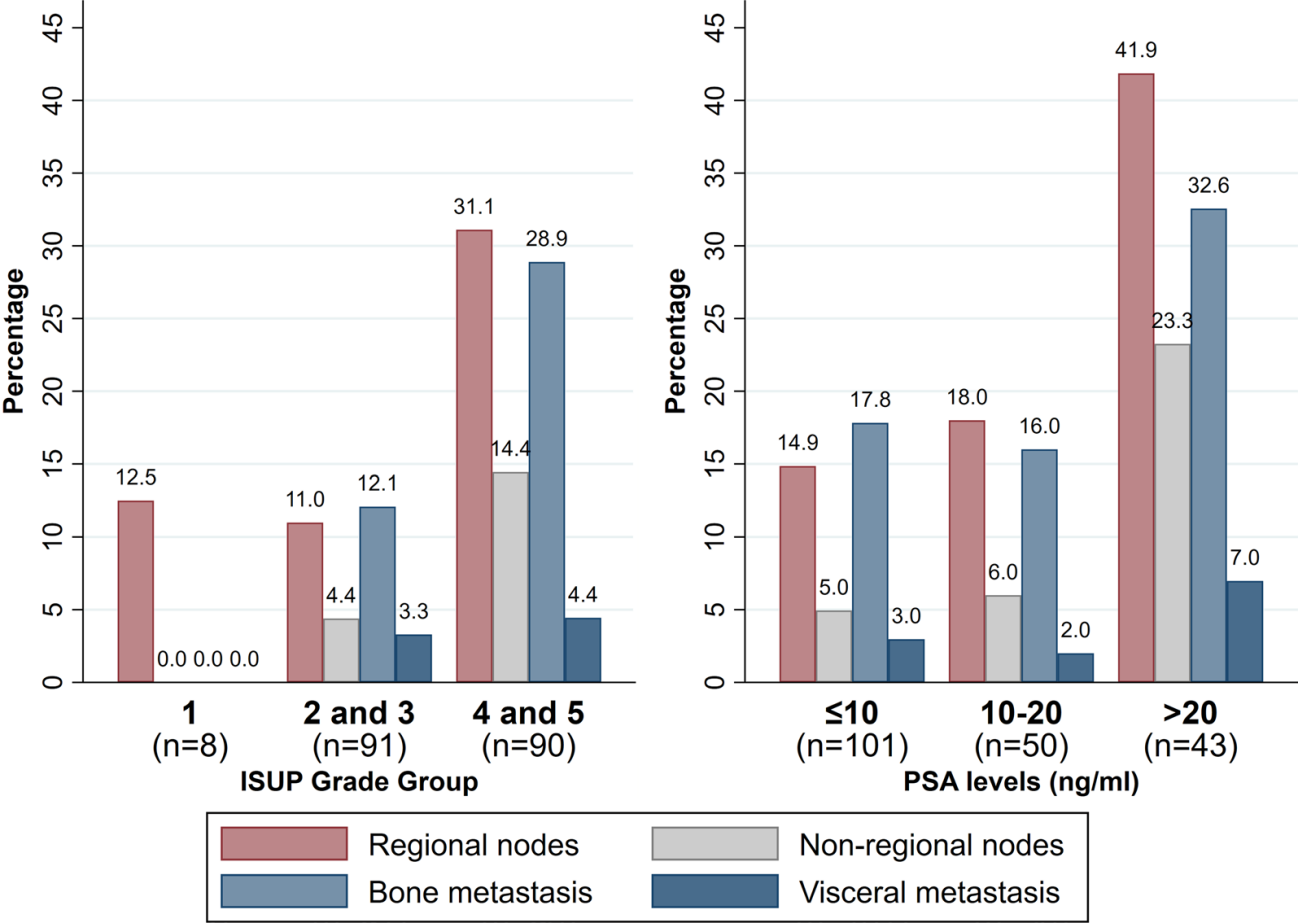
Bar graph demonstrating increase in involved metastatic regions and number of metastases with increasing ISUP grade and PSA levels on [^18^F]PSMA-1007 PET/CT (refer Tables 4 and 5)

Similarly, there was an increase in the number of regional and non-regional nodal metastases with increasing PSA levels. There were 14.9% (15/101) regional nodal metastases in PSA ≤ 10 ng/mL, 18% (9/50) in PSA 10–20 ng/mL and 41.9% (18/43) in PSA > 20 ng/mL group. The non-regional nodal metastases were 5% (5/101) in PSA ≤ 10 ng/mL, 6% (3/50) in PSA 10–20 ng/mL and 23.3% (10/43) in PSA > 20 ng/mL group. The PSA level was positively correlated with the presence of regional (*p* = 0.001) and non-regional nodal metastasis (*p* = 0.004), but not bone (p = 0.087) metastases. There were too few visceral metastases for meaningful analysis (*p* = 0.51) (Table 5). Overall, the number of involved metastatic regions increases with increasing PSA level and ISUP grade. This information has been provided in Tables 4 and 5 and plotted in Fig. 4.

**Table 5:** Association of PSA levels with SUVmax of the primary prostatic tumour and distribution of metastasis on [^18^F]PSMA-1007 PET/CT

	PSA levels (ng/mL)			*p*-value
	≤ 10	10–20	> 20	
	*n* = 101	*n* = 50	*n* = 43	
SUVmax of primary tumour (*n* = 187), median (IQR)^a^	13.9 (7.7–22.8)	17.4 (11.9–29.9)	21.5 (16.2–36.3)	< 0.001
Regional nodes (*n* = 194), %(n/Total)^b^	14.9% (15/101)	18.0% (9/50)	41.9% (18/43)	0.001
Non-regional nodes (*n* = 194), %(*n*/Total)^c^	5.0% (5/101)	6.0% (3/50)	23.3% (10/43)	0.004
Bone metastasis (*n* = 194), %(*n*/Total) ^b^	17.8% (18/101)	16.0% (8/50)	32.6% (14/43)	0.087
Visceral metastasis (*n* = 194), %(*n*/Total)^c^	3.0% (3/101)	2.0% (1/50)	7.0% (3/43)	0.51

^a^Kruskal–Wallis-test

^b^Chi-squared test

^c^Fisher’s exact test

### ISUP grade, PSA level and AJCC stage with SUVmax of primary prostate tumour

**Fig. 5:**
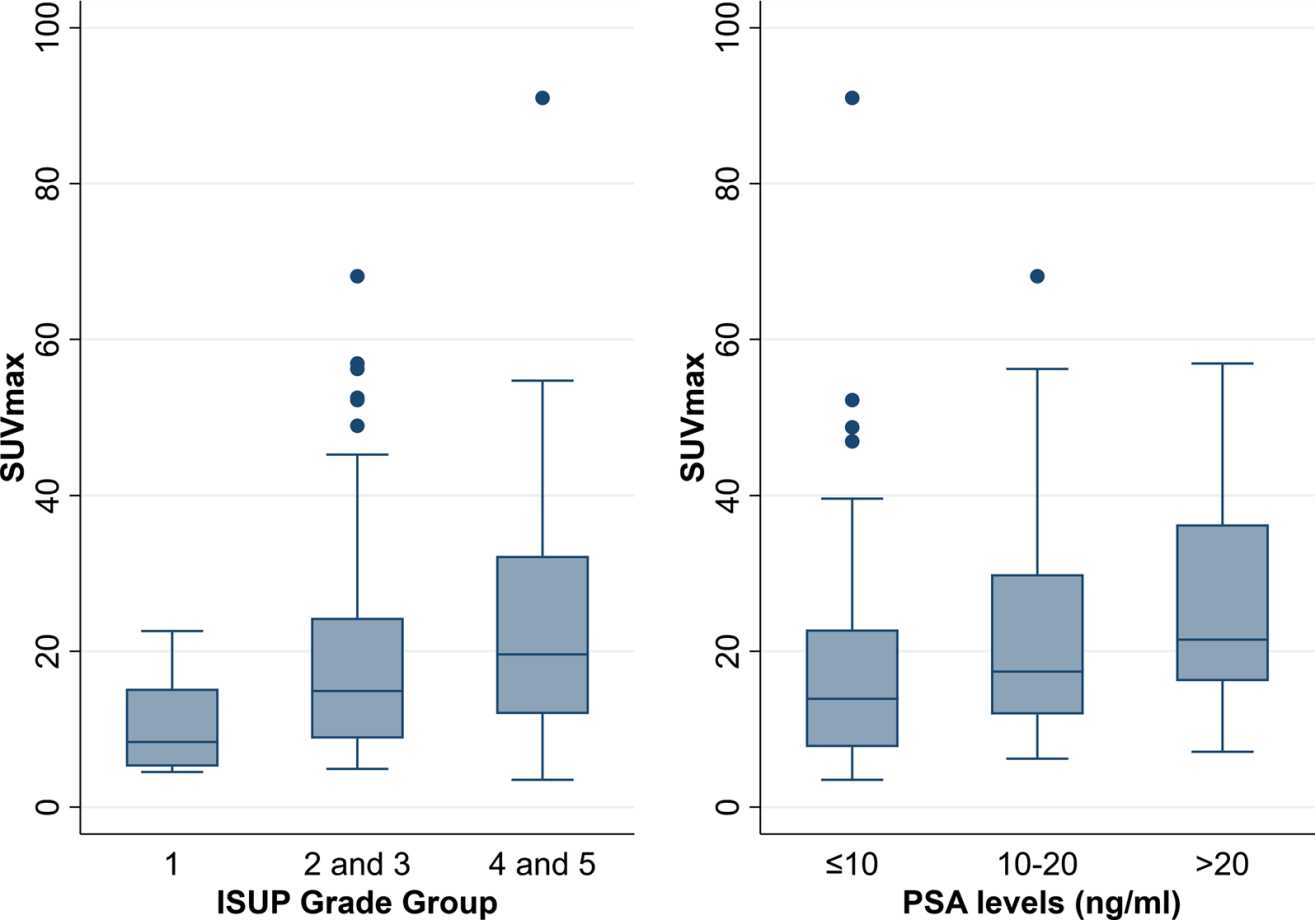
Box plot illustrates increasing primary prostate tumour SUVmax with increasing ISUP grades and PSA levels on [^18^F]PSMA-1007 PET/CT (*n* = 183)

There were seven negative [^18^F]PSMA-1007 PET/CT scans in the staging group that were excluded from the primary tumour SUVmax analysis (Additional file 1: supplementary table). The median SUVmax of the primary prostate lesion was 17.2 (IQR 9.5–26.7). The SUVmax of the primary prostate tumour was higher in the ISUP grades 4 and 5 compared to ISUP grades 2 and 3 (median SUVmax 19.6 vs 14.9). Similarly, the primary tumour SUVmax was higher in the PSA > 20 ng/mL group than PSA 10–20 ng/mL group (median SUVmax 21.5 vs. 17.4). The primary tumour SUVmax also increased with increasing AJCC stage with stage IIIB and below (median SUVmax 14.2), stage IIIC (median SUVmax 17.5), stage IVA (median SUVmax 19.8) and stage IVB (median SUVmax 20). A statistically significant association between ISUP grade (*p* = 0.004), PSA level (*p* < 0.001) and AJCC stage (*p* = 0.034) with SUVmax of the primary prostatic tumour (Tables 4, 5, 6) was observed. Additionally, a higher primary tumour SUVmax was detected in the high-risk group compared to the intermediate-risk group (median SUVmax 20.2 vs 11.9; *p* < 0.001). SUVmax of the primary prostate tumour with increasing ISUP grade and PSA levels is illustrated in Fig. 5 using box plots.

**Table 6:** Association of AJCC staging with SUVmax of the primary prostatic tumour (n = 187) on [^18^F]PSMA-1007 PET/CT

	AJCC stage				*p*-value
	IIIB or below	IIIC	IVA	IVB	
	*n* = 103	*n* = 27	*n* = 16	*n* = 48	
SUVmax of primary tumour (*n* = 187), median (IQR)^a^	14.2 (9.0–24.9)	17.5 (7.6–28.7)	19.8 (15.6–38.9)	20.0 (12.0–31.3)	0.034^a^

^a^Kruskal-Wallis-test

### Metastatic disease by EAU risk group

Overall, the high-risk group (PSA > 20 ng/mL or ISUP 4 and 5) had significantly higher proportion of patients with nodal (regional and non-regional) (34.7% vs 1.4%; *p* < 0.001) and bone (28% vs 8.5%; *p* = 0.001) metastases compared to the intermediate-risk group (PSA 10–20 ng/mL or ISUP 2 and 3). Although the high-risk group had a higher proportion of patients with visceral metastases compared to the intermediate-risk group (4.2% vs 2.8%), there were too few patients for meaningful analysis (*p* = 0.71) (Table 7).

**Table 7:** Proportion of metastases by EAU risk groups (intermediate-risk vs high-risk group based on PSA and ISUP grade) (*n* = 189) on [^18^F]PSMA-1007 PET/CT

	Intermediate-risk (PSA 10–20 ng/mL or ISUP 2 and 3)	High-risk (PSA > 20 ng/mL or ISUP 4 and 5)	*p*-value
	*n* = 71	*n* = 118	
SUVmax of primary tumour^a^	11.9 (8.4–21.1)	20.2 (12.0–32.5)	< 0.001
Nodal metastasis(regional and non-regional)^b^	1.4% (1)	34.7% (41)	< 0.001
Bone metastasis^b^	8.5% (6)	28.0% (33)	0.001
Visceral metastasis^c^	2.8% (2)	4.2% (5)	0.71

^a^Mann-Whitney U test

^b^Chi-squared test

^c^Fisher’s exact test; *n* = 2 with missing ISUP grade information and PSA < 20 ng/mL (undetermined risk group) and n = 3 with PSA < 10 and ISUP = 1(low risk) were excluded; % (*n*) = percentage of patients (number of patients)

### Evaluation of EAU criteria for detection of metastatic disease

The EAU criteria for classifying high-risk prostate cancer patients includes PSA > 20 ng/mL or ISUP grade > 3. PSA > 20 ng/mL had a specificity of 85% (95%CI 78–91%) and a sensitivity of 36% (95% CI 25–49%); whereas ISUP grade > 3 has a sensitivity of 62% (95% CI 49–74%) and specificity of 60% (95% CI 50–68%) for detection of metastatic disease using [^18^F]PSMA-1007 PET/CT in our population (Table 8). Our referral criteria of ISUP grade ≥ 3 has a sensitivity of 94% (95% CI 85–98%) and specificity of 36% (95% CI 27–45%) for detection of metastatic disease. Table 8 illustrates the sensitivity, specificity, positive predictive value, negative predictive value and accuracy of PSA > 20 ng/mL and ISUP > 3 for the detection of metastasis on [^18^F]PSMA-1007 PET/CT.

**Table 8:** Sensitivity, specificity, positive predictive value, negative predictive value and accuracy of PSA > 20 ng/mL and ISUP > 3 for the detection of metastasis on [^18^F]PSMA-1007 PET/CT

	Sensitivity	Specificity	Positive predictive value (PPV)	Negative predictive value (NPV)	Accuracy
PSA > 20 ng/mL	36% (95% CI 25–49%)	85% (95% CI 78–91%)	56% (95% CI 40–71%)	72% (95% CI 64–79%)	69% (95% CI 62%–75%)
ISUP > 3	62% (95% CI 49–74%)	60% (95% CI 50–68%)	43% (95% CI 33–54%)	76% (95% CI 66–84%)	60% (95% CI 53%–67%)

Twenty-three patients had three PSA values within 12 months before the PET/CT for calculating PSA doubling time and velocity. Out of these 23 patients, the group with metastases (*n* = 7) had higher median PSA velocity (12.5 vs. 4.8 ng/mL/year; *p* = 0.32) and lower median PSA doubling time (7.3 vs. 31 months; *p* = 0.23) compared to the patients without metastasis (*n* = 16).

### PSMA scan referral criteria

Out of the 194 patients included in the study, 31 patients did not meet our referral criteria but underwent [^18^F]PSMA-1007 PET/CT scan due to one of five reasons: investigation of high-risk radiological features or indeterminate lesion demonstrated on either CT, bone scan or MRI (15 patients), pretreatment scan after a period of active surveillance (5 patients), perineural invasion identified on biopsy (2 patients), high PSA level (PSA > 20 ng/mL) before the commencement of ADT (2 patients) and other/unknown reasons (7 patients). None of these scans were positive for metastatic disease.

### Receiver operating characteristic (ROC) curve

**Fig. 6:**
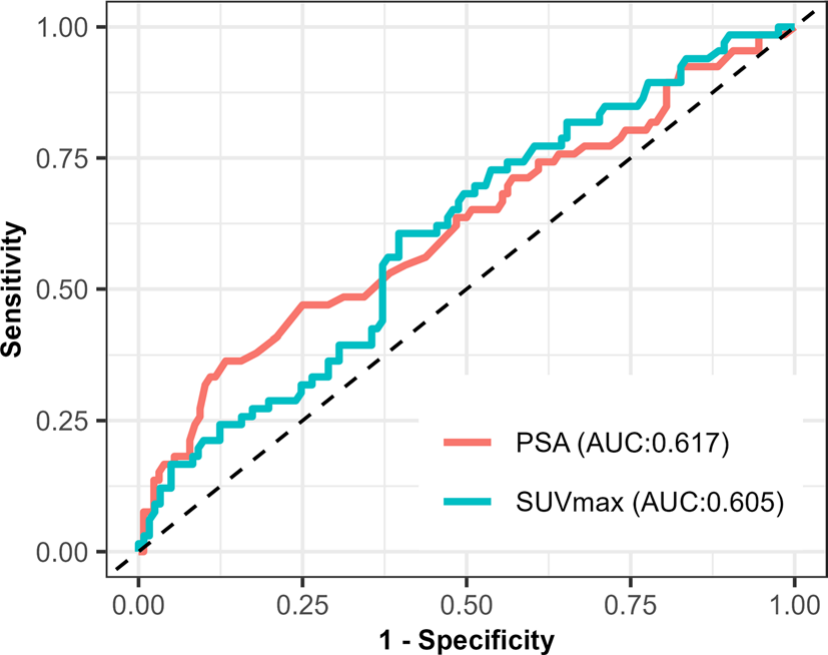
ROC curve of PSA and SUVmax of primary prostate tumour for predicting metastasis on [18F]PSMA-1007 PET/CT. Abbreviation AUC = Area Under the Curve

ROC curve analysis using PSA and SUVmax of the primary prostate tumour to predict metastasis on [^18^F]PSMA-1007 PET/CT is demonstrated in Fig. 6. The AUC (95% CI) for PSA and SUVmax of the primary prostate tumour were 0.62 (0.53–0.70) and 0.61 (0.52–0.69), respectively.

## Discussion

To our knowledge, this is the first study demonstrating a positive correlation between ISUP grade and PSA level with an increased likelihood of metastatic disease using [^18^F]PSMA-1007 PET/CT for primary staging of prostate cancer. Additionally, the distribution of metastasis was also analysed in our study. There was a strong positive correlation between PSA level and the likelihood of regional and non-regional nodal metastases and ISUP grade and regional, non-regional nodal and bone metastases. There was a trend toward increasing PSA level and bone/visceral metastases and ISUP grade and visceral metastases, without reaching statistical significance, potentially due to a small number of these cases. Furthermore, we demonstrate a higher proportion of patient with nodal (regional and non-regional) and bone metastases in the high-risk group compared to the intermediate-risk group. These results are concordant with the findings previously demonstrated using ^68^Ga-PSMA PET/CT [19, 21, 22], and whilst expected, these findings are an important step to validate the role of [^18^F]PSMA-1007 PET/CT in primary prostate cancer staging.

The presence of an unexpectedly high proportion of metastases in the PSA ≤ 10 ng/mL (15% regional nodal and 18% bone metastases) and ISUP grade 1 (13% regional nodal metastases) likely represents selection bias due to our PSMA PET/CT scan referral criteria requiring the presence of high-risk features for imaging (Tables 2, 3, 4, 5). For example, the one patient with regional nodal metastasis in ISUP grade 1 had a PSA level of 22 ng/mL placing them in the high-risk group. Similarly, the PSA ≤ 10 ng/mL group had a high proportion of intermediate-risk ISUP grades 2 and 3 (49%) and high-risk ISUP grades 4 and 5 patients (48%) (Table 2).

Ours is also the largest study to date using [^18^F]PSMA-1007 PET/CT confirming a positive correlation between higher ISUP grade and PSA level with increasing SUVmax of the primary prostate tumour, consistent with increasing PSMA receptor expression (Fig. 5). Further, a higher primary tumour SUVmax was confirmed in the high-risk group compared to the intermediate-risk group. Two smaller [^18^F]PSMA-1007 studies [33, 34] and a similar [^68^Ga]Ga-PSMA-11 study [35] have shown a similar correlation with higher SUVmax of the primary prostate tumour with increasing PSA levels and Gleason score/ISUP grade. In cases when a prostate biopsy cannot be performed due to patient preference or contraindications, the SUVmax of the primary tumour on the [^18^F]PSMA-1007 PET/CT may be indirectly used to estimate ISUP grade with higher SUVmax values implying a likelihood of a high ISUP grade. This can be confirmed in prospective studies. Additionally, there was a statistically significant correlation between increasing primary prostate tumour SUVmax with increasing AJCC stage, with similar implications.

Lastly, our study evaluated the performance of EAU high-risk parameters (PSA level > 20 ng/mL and ISUP grade > 3) in detecting metastatic disease using [^18^F]PSMA-1007 PET/CT in our selected population. We found that PSA > 20 ng/mL has a good specificity of 85% (sensitivity of 36%) for the identification of metastatic disease using [^18^F]PSMA-1007 PET/CT (Table 8). However, ISUP grade > 3 only has a sensitivity of 62% (specificity of 60%), whereas our referral criteria of ISUP ≥ 3 has a much greater sensitivity of 94% (specificity 36%) for detecting metastatic disease using [^18^F]PSMA-1007 PET/CT (Table 8). Therefore, using PSA > 20 ng/mL or ISUP ≥ 3 as selection criteria would be adequate for identifying most patients with metastatic disease using [^18^F]PSMA-1007 PET/CT for prostate cancer staging. The current EAU guidelines use similar parameters to recommend imaging, albeit conventional imaging, for staging prostate cancer [17].

There are a few limitations to our study. Firstly, given the study's retrospective nature, data collection was incomplete for some patients (missing information on PSA levels, ISUP grade and treatment information) potentially introducing bias and loss of statistical power, possibly avoidable in future prospective studies. Secondly, similar to other published [^18^F]PSMA-1007 PET/CT studies, typical focal prostatic and extraprostatic avidity was considered disease without histopathological confirmation. It is not feasible to pathologically confirm every disease site due to patient factors, location or small lesion size. However, sufficient evidence now exists to use [^18^F]PSMA-1007 PET/CT as a standard of reference and a substitute for histopathological validation, as explained in the methods section [4, 13, 14, 26–30]. Thirdly, a common issue with the [^18^F]PSMA-1007 radiotracer is focal uptake in non-specific bone lesions (NSBLs) without an underlying CT correlate definite for prostate metastasis. Recent data suggest that these NSBLs rarely represent metastatic lesions, supporting our image interpretation that equivocal lesions are not considered malignant [36]. Lastly, a small number of patients were on ADT before the PET/CT scan during initial staging, presumably due to high-risk features, potentially influencing the PSMA targeted PET/CT results [37].

## Conclusion

Our study confirms PSA level and ISUP grade as significant prognostic risk factors for the presence and distribution of metastatic disease using [^18^F]PSMA-1007 PET/CT for primary staging of prostate carcinoma. Furthermore, this is the largest study confirming a positive correlation of PSA level, ISUP grade and AJCC stage with primary prostate tumour SUVmax using [^18^F]PSMA-1007 PET/CT. This study also demonstrates adequate performance of PSA > 20 ng/mL or ISUP grade ≥ 3 parameters for the detection of metastatic disease using [^18^F]PSMA-1007 PET/CT. Validation of these findings further supports the use of [^18^F]PSMA-1007 PET/CT for the primary staging of prostate carcinoma.

## Supplementary Information

**Additional file 1**. Supplementary table lists the patients' age, Gleason score, ISUP grade, histology and PSA level for the seven patients with negative [18F]PSMA-1007 PET/CT.

## Data Availability

Not applicable.
