# Supplementary material for: Risk of metastatic disease using [18F]PSMA-1007 PET/CT for primary prostate cancer staging
Source: EJNMMI Res. 2021 Dec 20;11:128. doi: 10.1186/s13550-021-00869-5 (PMC8688644; doi:10.1186/s13550-021-00869-5)
Supplement: Supplementary file 1 — Additional file 1. Supplementary table lists the patients' age, Gleason score, ISUP grade, histology and PSA level for the seven patients with negative [18F]PSMA-1007 PET/CT. [file 13550_2021_869_MOESM1_ESM.docx]

# Supplementary material

# Title: Risk of metastatic disease using [^18^F]PSMA-1007 PET/CT for primary prostate cancer staging

**Journal Name: European Journal of Nuclear Medicine and Molecular Imaging**

**Authors:** Venkata A. Chikatamarla^1,2^, Satomi Okano^3^, Peter Jenvey^4^, Alexander Ansaldo, Matthew J. Roberts^5,6,7^, Stuart C. Ramsay^1,8^, Paul A. Thomas^1,2^, David A. Pattison^1,2^.

1. Department of Nuclear Medicine & Specialised PET Services, Royal Brisbane & Women’s Hospital, Brisbane, Australia
2. Faculty of Medicine, University of Queensland, Brisbane, Australia
3. Statistics Unit, QIMR Berghofer Medical Research Institute, Brisbane, Australia
4. Department of Medical Imaging, Royal Brisbane & Women’s Hospital, Brisbane, Australia
5. Department of Urology, Royal Brisbane and Women’s Hospital, Brisbane, Australia
6. Department of Urology, Redcliffe Hospital, Redcliffe, Australia
7. The University of Queensland, Centre for Clinical Research, Brisbane, Australia
8. School of Medicine, James Cook University, Townsville, Australia

**Corresponding & First Author:**

Dr Venkata A. Chikatamarla

Department of Nuclear Medicine & Specialised PET Services

Royal Brisbane & Women’s Hospital

Brisbane, Queensland, Australia 4006

T: +61450893007

Email: [cvavinash@gmail.com](mailto:cvavinash@gmail.com)

ORCID: 0000-0002-8996-9146

**Supplementary table**

**Table 1** Seven patients with negative [^18^F]PSMA-1007 PET/CT

| **Patient Number** | **Age** | **Gleason Score** | **ISUP grade** | **Histology** | **PSA level (ng/mL)** |
| --- | --- | --- | --- | --- | --- |
| 1 | 83 | Unknown | Unknown | Unknown | 12 |
| 2 | 63 | 3+3=6 | 1 | Acinar adenocarcinoma | 17 |
| 3 | 73 | 3+3=6 | 1 | Acinar adenocarcinoma | 22 |
| 4 | 62 | 3+4=7 | 2 | Acinar adenocarcinoma | 6.9 |
| 5 | 65 | 3+4=7 | 2 | Acinar adenocarcinoma | 9.6 |
| 6 | 59 | 4+5=9 | 5 | Acinar adenocarcinoma | 13 |
| 7 | 82 | 4+5=9 | 5 | Acinar adenocarcinoma | 2.1 |
